# Surgical Matters—Ovariotomy

**Published:** 1859-05

**Authors:** 


### MISCELLANEOUS MEDICAL INTELLIGENCE.

### SURGICAL MATTERS—OVARIOTOMY.

Prof. Pope, of St. Louis, has recently reported four cases of ovariotomy, 2 fatal and 2 recovered.

Prof. Hamilton, of Columbus, reports 2 cases; 1 successful, 1 fatal.

Prof. H. states, that of 24 cases which have come to his knowledge, of operations performed in Ohio, 13 were fatal and 11 successful.

Dr. W. L. Atlee, in 1851, reported to the American Medical Association a synopsis of 222 cases of ovariotomy. Of these,

| | Recoveries. | Deaths. |
|---|---|---|
| 52 were of the minor section, | 39 | 13 |
| 153 " " major " | 95 | 58 |
| 17 Unknown, | 12 | 5 |
| | 146 | 76 |

Of the 222 cases, 57 could not be finished—of these, 45 recovered, 12 died.

The Academy of Medicine, in Paris, discussed the subject of ovarian cysts in 1856 and 1857. They were almost unanimous against ovariotomy, Cazeau alone being in favor of it.

They were equally unanimous in favor of injections of iodine, although admitting that it could only be successful in the unilocular form.

Prof. Miller, of Louisville, recently published an excellent article in the *American Journal*, which seems to be intended as a reply to the attack made in the Academy on the operation. The views of Prof. Miller are eminently just. He has himself performed the operation twice, with success. Still there are three things to be said in palliation of the extreme view taken by the Academy:

1. The operation has been abused: a number of cases having been operated, where it was easy to have determined that no tumor existed.

2. The constitution of the French and other women on the Continent are not as robust as that of the women of this country. This is owing, in part, to the nourishment of the laboring classes there being insufficient, and in part to a cause little suspected, viz: that animal life is less vigorous there than here. We have noticed this in regard to frogs, pigeons, rabbits, etc., which we have used for experiments in both places, and Dr. Brown-Sequard confirms this view.

3. Since the mania for ovariotomy has become so pressing, the palliative means, and particularly the injections of iodine, have been entirely neglected. This is an error. In cases of single cyst it is a safe and efficient remedy.

Prof. Miller attributes the performance of this operation to Dr. Ephraim McDonnell, of Ky., in Dec., 1809. This, if correct, is a national honor which ought not to be forgotten.

